# New Clinical Applications of Electrolyzed Water: A Review

**DOI:** 10.3390/microorganisms9010136

**Published:** 2021-01-08

**Authors:** Pianpian Yan, Eric Banan-Mwine Daliri, Deog-Hwan Oh

**Affiliations:** Department of Food Science and Biotechnology, Kangwon National University, Chuncheon 24341, Korea; pianpianyan1@gmail.com (P.Y.); ericdaliri@yahoo.com (E.B.-M.D.)

**Keywords:** electrolyzed water, clinical application, antimicrobial effect, wound healing, antibiofilm, oral hygiene

## Abstract

As the situation of severe acute respiratory syndrome coronavirus type 2 (SARS-CoV-2) is still deteriorating, there has been a huge increase in the demand and use of disinfectants. Electrolyzed water (EW), as a novel broad-spectrum disinfectant and cleaner, has been widely used for several years. EW can be produced in an electrolysis chamber which contains dilute salt and tap water. It is an effective antimicrobial and antibiofilm agent, with several advantages such as on-the-spot, cheap, environmentally friendly and safe for human beings. Therefore, EW holds potential significance for high-risk settings in hospitals and other clinical facilities. EW can also be applied for wound healing, advanced tissue care, and dental clinics. The present review article highlights the latest developments and new perspectives of EW, especially in clinical fields. Furthermore, the main action modes of antibiofilm and antimicrobial will be summarized.

## 1. Introduction

The Center for Disease Control and Prevention (CDC) has recently reported that there is at least one person who has a healthcare-associated infection in every 31 hospital patients in any given day [1]. Such healthcare-associated infections (HAI) include central line-associated bloodstream infection, catheter-associated urinary tract infections, surgical site infection and ventilator-associated pneumonia [2]. HAIs are a major cause of morbidity and even mortality in the United States [3]. The healthcare environment is a primary source of pathogenic microorganisms [4]. Molds may be present on wet or damp surfaces or materials [5]. Bacteria may also be present in bathroom installations, including sink drains and ice machines. Furthermore, surgical site infections can sometimes be superficial infections involving the skin [6,7]. At the same time, infections in other surgical sites could be more serious, which may involve tissues under the skin, organs, or even implanted materials [8,9]. Infections also increase the length of stay, readmission rates, costs, and even mortality [10,11]. Biofilms are responsible for causing 80% of human infections. The National Institutes of Health (NIH) reported that biofilms are responsible for up to 80% of human bacterial infection [12].

Therefore, developing effective disinfectants and antiseptics for killing pathogens and destroying the biofilm formation in the environment and human healthcare is one of the most significant steps for infection prevention and control. The medical industry has employed a number of decontamination techniques throughout the hospital and healthcare clinical field [13,14,15]. However, some of these techniques have disadvantages such as high cost, low efficacy, remaining chemical residues, and adverse effects irritation on the human skin [16,17]. As an important premise for practical application, it should have high antimicrobial efficacy and no toxicity to the human body [18].

Electrolyzed water (EW) is a novel disinfectant and cleaner which has been widely used in the food industry for several years to ensure the sterilization of surfaces and safety of food [19,20,21,22]. EW is produced in an electrolysis chamber which contains dilute salt and tap water without any harmful chemical addition [23]. EW has antimicrobial effects against a variety of microorganisms including common biofilm, viruses, bacteria, spores and fungi in chronic wounds and environmental surfaces [24,25,26,27,28,29]. Currently, due to its beneficial properties (anti-infection and cell proliferative), researchers pay more attention to the application of electrolyzed water in clinical treatments including medical sterilization. The US Environmental Protection Agency (EPA) recommended the use of disinfectants with hypochlorite acid as active ingredients for the disinfection of surfaces against COVID-19 [30]. Furthermore, various studies have been carried out on the antimicrobial activity of EW against different illments, including diabetic foot ulcers [31,32], venous ulcers in the legs [33,34] or feminine hygiene [35,36].

However, some studies have reported that the application of EW is limited by factors such as the corrosion of equipment which is in contact with acidic or basic EW and the ability of organics materials (proteins, lipids and so on) to shorten its shelf life [37,38]. To overcome these defects, hurdle technology, which is a combination of two or more low-dose disinfection and preservatives techniques could be applied [39]. Therefore, EW combined with other disinfection methods could be an effective way to obtain a desirable result [40,41].

The aim of this review was to introduce recent developments and provide a new perspective with EW in the clinical field. Many characteristics of electrolyzed water in this review article were introduced including the physiochemical properties, history, limitation principle, generation methodologies, and the impact of these characteristics on the sanitizing efficacy of EW. In addition, applications of EW for microbial control in the clinical field are also discussed.

## 2. Principles and History of EW

**Figure 1:**
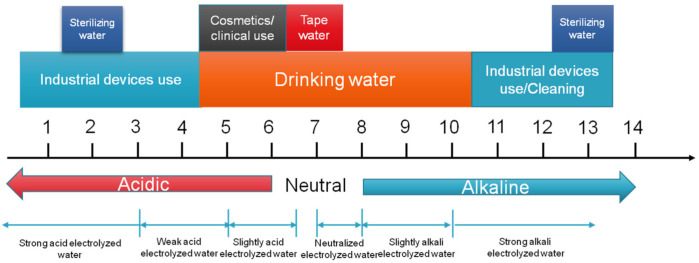
Application of electrolyzed water (EW) at different pH values in various fields.

The development history of electrolyzed water can be traced back for more than a century [42]. The concept of electrolyzed water was first proposed in Russia [43]. However, it has been widely used for various purposes including disinfection, water regeneration and water decontamination in Japan since 1980. As time went by, its application has extended to other fields such as the food industry, agriculture, livestock management and clinical application [44,45,46,47]. Figure 1 illustrates the application of EW in different areas at different pH values.

Electrolyzed reduced water was invented in the early 19th century [48]. Research on electrolyzed water started in Japan around 1931 and its application and popularity to agriculture in the 1950s. In 1960, the water was applied to medical care and in 1966, electrolyzed reduced water was touted as having “healing effects” including indigestion, chronic diarrhea, antacid, abnormal gastrointestinal fermentation, and hyperacidity [49]. A device for the preparation of ERW was authorized for home-use by the Ministry of Health, Labor, and Welfare of Japan [50]. 

In 1994, with the support of the Ministry of Health, Labor, and Welfare of Japan, the functional water foundation was established to promote the use of electrolyzed water in society. Based on considerable scientific evidence related to the risk assessment of EW, in 2005, the Drugs, Cosmetics and Medical Instruments Act of Japan was revised and re-authorized an ERW-producing device as a home-managed medical device. In 2002, the Ministry authorized the use of hypochlorous acid water on designated food additives. Recently, in 2017, the US Food and Drug Administration (USFDA) also authorized hypochlorous acid (electrolytically generated on-site) for use on food contact surfaces (FCS) [51]. In addition, Chinese standardization administration published a series of criteria in 2020, related to hypochlorous acid water, which can be used for human skin, hand and mucous membrane. Table 1 illustrates the criteria of application of EW in different countries.

## 3. Systems for Generation of Electrolyzed Water

Electrolyzed water (EW) is produced in an electrolysis chamber which contains hydrogen chloride (HCl) solution or dilute salt (NaCl) [52]. According to the different devices, electrolyte and electrolysis conditions, EW can be classified into the following categories: acidic electrolyzed water, neutral electrolyzed water and alkali electrolyzed water [53]. The characteristic of EW is shown in Table 2. The application of EW can be roughly divided into alkali water for drinking and electrolytic water for cleaning, sterilization, and disinfection [49,54,55,56].

**Figure 2:**
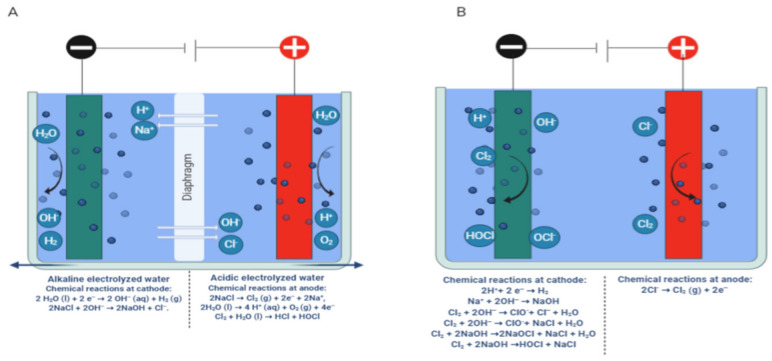
Generation of electrolyzed water. (**A**): alkaline electrolyzed water and acidic electrolyzed water; (**B**): slightly acidic electrolyzed water. Created with BioRender.com.

These solutions are produced by the electrolysis of dilute salt (NaCl) passing through two or three cell electrolyzers with the anode and cathode separated by a diaphragm. It can produce two types of water simultaneously. Acidic electrolyzed water (AEW), with a pH of 2 to 3, available chlorine concentration (ACC) of 10 to 90, and oxidation–reduction potential (ORP) >1100 Mv, is produced at the anode side [23]. At the same time, basic electrolyzed water (BEW) with a pH of 10 to 13, and ORP from −800 to −900 Mv is generated at the cathode side. Nowadays, there are some novel forms of electrolyzed water such as slightly acid electrolyzed water (SAEW), weak acid electrolyzed water (WAEW) and neutral electrolyzed water (NEW) [57,58,59]. SAEW is very popular in Japan, China and Korea [60,61,62]. SAEW (pH of 5.5–6.5, ACC of 10–80 ppm and ORP of 800–900 Mv), and NEW (pH of 7–8 and ORP of 750–900 Mv) are produced by using single-cell chambers. SAEW is produced by the electrolysis of HCl alone or combined with NaCl in a single-cell unit (without diaphragm) [63]. It is expected that the SAEW will not lose its superior features after mixing due to the unipolar reaction in the process of electrolysis. In addition to the above method, NEW can also be produced by a mixture of the anodic solution with OH^−^ ions [64]. The details are shown in Figure 2. EW can also be stored in containers of special materials or converted into ice cubes for future use [65].

## 4. Factors Influencing Decontamination Efficacy of Electrolyzed Water

### 4.1. Direct Factors

**Figure 3:**
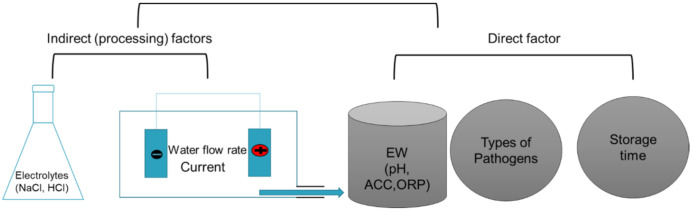
Factors affecting the decontamination efficacy of electrolyzed water. ACC: available chlorine concentration; ORP: oxidation–reduction potential.

The concentration of chlorine (Cl_2_, OCl^−^, and HOCl), ORP, and pH directly play an important role in the antimicrobial efficacy of EW (shown in the Figure 3). HOCl is the most effective inactivation compound in the chlorine group [66]. They found that the inactivation efficacy of HOCl was 80-fold higher than that of an equivalent concentration of OCl^−^ when the pH value of the solution was from 5.0 to 6.5. Ding et al. reported that SAEW treatment on *S. aureus* for 1 min reduced 5.8 log CFU/mL, but sodium hypochlorite (NaClO) decreased by the bacteria by 3.26 log CFU/mL [67]. This might be explained by considering that the electrical properties of the HOCl and OCl^−^ are different. HOCl is neutral, whereas the hypochlorite ion (OCl^−^) and bacterial membrane are both negative [68]. Therefore, HOCl can more easily penetrate target cells to exert strong bactericidal effects based on Coulomb’s law. However, the fraction of chlorine species depends on the pH of the solution [69]. HOCl is a weak acid with a pKa of about 7.46 [70]. Therefore, if the pH value is low (pH < 4), it is possible to form Cl_2_. When the pH value is above 7.5, HOCl is decomposed into hydrogen ion (H^+^) and hypochlorite ion (OCl^−^) in the reversible reaction [70]. HOCl, as one of the reactive oxygen species (ROS), infiltrates the membranes of bacteria cells and kills pathogens through chlorination or oxidation, which destructs the key metabolic frameworks [71]. In addition, there are a few reports of the inactivation action being mainly affected by the ORP of EW. They reported that high ORP may result in modifying the metabolic flux and ATP production [72]. Liao et al. studied the inactivation mechanism of ORP in EOW. The results showed that EOW with higher ORP had a higher efficiency of the inactivation of E. coli O157:H7 by damaging the outer membrane and inner membrane, thus releasing the intracellular component [73]. 

### 4.2. Indirect Factors

The concentration of electrolyte, water flow rate and water source (hardness) indirectly influence the effectiveness of EW(shown in the Figure 3). However, the above factors are linearly correlated to the amount of HOCl and ORP in the process of electrolysis and ultimately reduce or increase the decontamination efficacy of EW (the properties of EW). 

Kim et al. [26] examined the effects of the water hardness of SAEW in inactivating *Staphylococcus aureus*, *Salmonella enterica serovar Typhimurium*, *Escherichia coli*, and *Bacillus cereus* spores. The results showed that the ACC of SAEW produced by tap water (hardness = 29 ppm) is better than that of underground water (hardness = 12 ppm). The hardness of water is mainly dependent on the content of calcium and magnesium [74]. There is a positive correlation between salinity and conductivity. In addition, electrical conductivity and the total chlorine concentration of the electrolyzed oxidizing water increased with the increasing salt concentration. When the concentration of salt (KCl) was increased from 2.0 M to 3.0 M, the ACC increased from 56.5 to 65.5 ppm in the same time [26]. 

Moreover, the water flow rate affects the ACC. Hsu et al. reported that the total ACC and ORP of electrolyzed oxidizing water was significantly decreased when water flow rate and salt concentration increased in the feed solution [75]. The reasons are maybe that the higher flow rate leads to less residence of ions in the electrolysis cell per unit time, chloride ions and sodium ions could not be sufficiently electrolyzed and moved to the anode side [74]. Therefore, more sodium, chloride ions and less HOCl remained in the feed water.

## 5. The Advantages and Disadvantages of Electrolyzed Water

There are many advantages of EW over its toxic counterparts (physical, chemical and biological technology) in different areas such as agriculture, food hygiene, medical field and even in human surface disinfection. The advantages of electrolyzed water can be easily enumerated. 

First, EW has been proposed as an environmentally friendly alternative to physical and chemical methods, which do not contain undesirable toxic contaminants [76]. As previously mentioned above, EW is only produced from NaCl and tap water and reverts to regular water after use [77]. Second, EW has a broad-spectrum inactivation ability and rapid antibacterial activity, which possesses nonselective properties [78,79]. HOCl was produced by an enzyme called myeloperoxidase, which uses hydrogen peroxide (H_2_O_2_) in our body as a substrate to react with neutrophils. [80]. HOCl is a naturally occurring molecule and has strong bactericidal ability to serve as a reliable defense system [78]. Medina et al. reported that artificially contaminated eggs with *Salmonella* or *E. coli* reduced >1.45 Log10 CFU/egg and >6.39 Log10 CFU/egg, respectively, after 30 s treatment of NEW [72]. Third, EW-producing machines have the ability for on-site generation at the location of intended use inexpensively [23]. The volume of 1 L of EW can be made in 8 min and the process can be repeated multiple times a day [19]. Therefore, it can prevent chlorination problems during handling, storage, and transport. Additionally, the use of AEW, alkaline electrolyzed water (AlEW), NEW, and SAEW do not cause negative organoleptic changes in food [49,81,82]. Finally, NEW and SAEW have a neutral pH and are safe, with no irritation on mucous membrane and skin [83].

When tackling the disadvantages and advantages of EW, we also need to point out the adverse impact of this novel technology. First, EW is a sanitizer produced from tap water with sodium chloride (NaCl) without the addition of harmful chemicals [84]. However, it still contains chemical compounds. The USFDA published a regulation that when EW is used to process fruits, vegetables, ready-to-eat meats, fish and seafood products intended to be consumed raw, the treatment will be followed by either a 10 min drain step or a potable water rinse to remove residues [51]. In addition, the Ministry of Health, Labour and Welfare (Japan) issued an act to remove HOCl before it becomes the final product. Second, the concentration of chlorine decreases over time, and loses its antimicrobial potential quickly [85]. Third, the degradation of synthetic resins and metal corrosion can be caused by high ORP or the free chlorine content during the use of AEW [22,86]. 

## 6. Disinfection Mechanisms of EW

**Figure 4:**
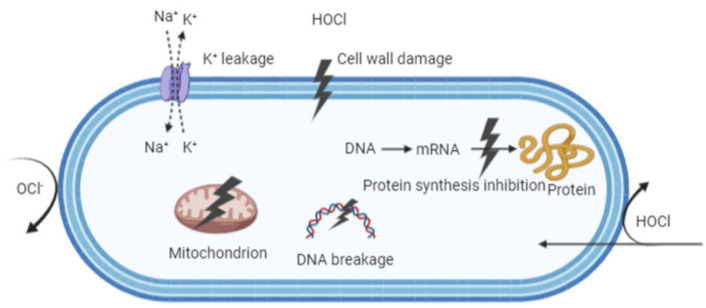
Model representing the mechanism of electrolyzed water. Created with BioRender.com.

In order to produce the safe and effective use of disinfectants, numerous disinfection methods have been studied and reported over the years. Many researchers have fully studied the mechanism of traditional disinfection methods such as physical treatments (heat and irradiation etc.) and chemical disinfectants (hydrogen peroxide and chlorine dioxide etc.) [87]. However, the exact mechanisms underlying microbial inactivation by EW have not been fully elucidated. It is well known that chlorine (Cl_2_, −OCl, and HOCl) plays an important role in the antimicrobial efficacy of electrolyzed water [88]. HOCl can penetrate the lipid bilayer of the cell membrane by passive diffusion due to its molecular size (which is equivalent to water (H_2_O)) and its electrical neutrality [89]. In addition, HOCl is a powerful oxidizing agent, which denatures and aggregates proteins [90]. These may be the reason for the excellent germicidal activity of HOCl. Ding et al. found that SAEW disrupted cell membrane permeability by damaging membrane proteins, entering the cells and causing the agglutination of cellular inclusions in *S. aureus* [67]. Furthermore, Tang et al. reported that EOW decreased the activity of TCC-dehydrogenase, intensified the permeability of the membrane, increased the conductivity of suspension, and resulted in the leakage of K^+^, protein and DNA, which indicated that the cell wall and membrane were damaged [91]. However, OCl^–^ cannot penetrate the microbial cell and microbial membrane because there is a lipid bilayer in the plasma membrane (hydrophobic layer) [92]. OCl^−^ only exhibits an oxidizing action from outside the cell, which would inactivate functional proteins localized in the plasma membrane [93]. In addition to the chlorine family, other compounds (reactive oxygen species) can be produced in the process of electrolysis, which contributes to the antimicrobial efficiency [94]. Figure 4 shows the mechanism of HOCl and OCl^–^ reaction on pathogens. The exact pattern of EW on microbial cells is still unclear and requires more investigations to clarify in the future.

## 7. Use of EW for Clinical Application

Recently, the Ministry of Health of the People’s Republic of China released three Chinese standards for materials and restricted substances in disinfectants, general requirements for hand disinfectants and general requirements for the disinfectants of mucous membrane in April 2020 [95,96]. In short, EW can not only be used for disinfecting medical instruments, clinical environments and object surfaces, but also disinfecting hands, skin, and mucous membranes. In addition, the US Environmental Protection Agency has recommended many disinfectants for COVID-19, including HOCl. Currently, there are a variety of EW-based disinfection products on the market. The approved core formula is HOCl, which can remain stable for up to twelve months without cytotoxicity [97]. Importantly, its pH neutralization can enhance therapeutic activity, stability and skin tolerability. Many patents including the use of EW application for advanced tissue care, dermatology and dental care are available [98,99,100]. The application of EW in the clinical field was shown in Table 3.

### 7.1. Wound Care

**Figure 5:**
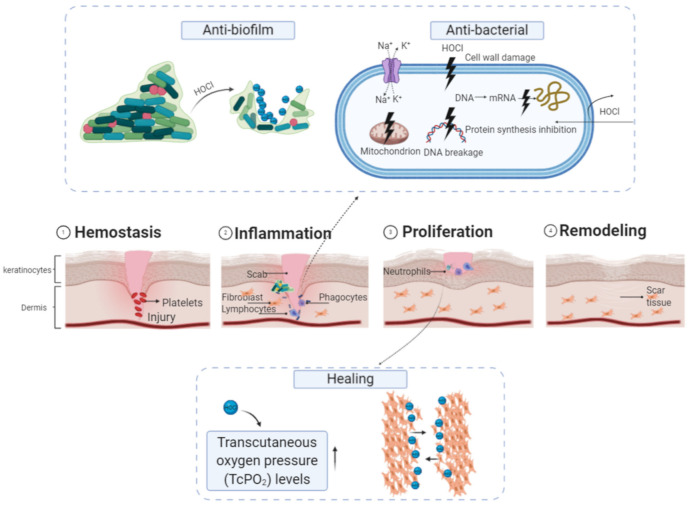
Model representing the mechanism of electrolyzed water on wounds. Created with BioRender.com.

A topical antibacterial agent, which can reduce the bacterial biological load of the wound without impairing the healing ability, is an imperative condition for therapy [124]. Wound healing is a complex process including multiple stages: hemostasis, inflammation, proliferation and tissue remodeling [125]. The timely resolution of each healing process is critical for promoting healing and avoiding excess scar formation. Currently, the treatments for impaired wound healing focus mainly on the optimization of controllable factors including the clearance of infections, mechanical protection, and nutritional support [126]. Wound care should also minimize scarring and inflammation. Recently, EW with antimicrobial properties has been utilized as part of cell proliferation, anti-infection and anti-biofilm therapies in a wound healing agent (shown in the Figure 5) [114,127]. Ben et al. found that with the application of MicroSafe^®^ as an instillation fluid with a novel foam dressing and negative pressure wound therapy for the patient, the wound bed showed dramatic improvement after three days of treatment [128]. Sasai et al. also studied the potential use of AEW for patients with atopic dermatitis. Their results also revealed that the treatment with 3 min spraying and after 1 week of skin reduced the *Staphylococcus aureus* count by about 3.80 log/cm^2^ reduction without any detrimental effect [108]. Scientists reported that electrolyzed water has an effect on skin wound healing. Tiroda et al. reported that nine patients (23%) using superoxidized solution improved by at least 75% in the reduction in lesions [129]. Additionally, biofilm formation causes prolonged wound infections due to the dense biofilm structure, differential gene regulation to combat stress, and the production of extracellular polymeric substances [112]. HOCl (active compound) is able to increase oxygenation (TcPO_2_) in wounds while breaking biofilms, which is an important key differentiator from other products [130].

### 7.2. Hand Sanitizer

Hand sanitization is the most important but simple way to remove germs, prevent the spread of germs to others and avoiding illness [131]. For EW-based hand sanitizers sold in China, the concentration of ACC usually ranges from 30 to 150 ppm, which is effective against viruses and bacteria. In addition to using EW as a liquid-based disinfectant, EW in fog form also show an antibacterial effect against numerous types of bacteria [123]. Pathogens related to hand hygiene and healthcare include *Escherichia coli*, *Pseudomonas aeruginosa*, *Staphylococcus aureus*, *Enterococcus hirae*, *Candida albicans.* Sipahi et al. reported the inactivation effect of StAEW, SAEW, mixed electrolyzed water (MEW) and catholyte (CEW) on *Escherichia coli*, *Pseudomonas aeruginosa*, *Staphylococcus aureus*, *Enterococcus hirae*, *Candida albicans*. They found that StAEW, SAEW, and MEW reduced the agents significantly. StAEW was especially effective against test microorganism (*Pseudomonas aeruginosa, Enterococcus hirae* and *Candida albicans)* populations, which all decreased by 100% in 1 min [132]. HOCl (~95%) is the main compound of the active chlorine family in SAEW, which is considered to be the cause of microbial inactivation [133]. SAEW with a neutralized pH has attracted more and more attention as an antibacterial solution. SAEW may be a promising novel clinical disinfectant that may be considered as an alternative to traditional alcohol-based hand sanitizer [40,134].

### 7.3. Oral Hygiene

The dental community has long sought for appropriate antibacterial products to try to control and prevent the proliferation of oral microbiome, especially during dental surgery when host barrier function is often impaired. Microorganisms related to oral hygiene include *Streptococcus salivarius*, *Staphylococcus aureus*, *Lactobacillus casei*, *Aggregatibacter actinomycetemcomitans* [135,136,137,138]. They found that AEW significantly inhibited the above bacterial growth for 30 secs without negative cytotoxic effects [115]. Hsieh et al. studied the electrolyzed oxidizing (EO) water as a mouthwash against *Streptococcus mutans*. The results revealed that EOW (125 ppm) showed antimicrobial effectiveness (>99.9%) against *S. mutans* after soaking treatment for 3 min [139]. The contamination of the dental water unit line is one of the major causes of oral infection [140,141]. A study on the treatment of the oral comprehensive treatment station containing mouthwash and pipe water in hospitals by SAEW treatment has been reported. The qualified rate of water sanitation quality in the water treatment channel increased from 8.85 to 49.15 % [120]. Nakano et al. also reported that there was little negative effects concerning the use of SAEW for the water line of dental units during seven years of clinical trials [142].

### 7.4. Environmental Decontamination

Experts generally agree that the daily careful cleaning and/or disinfection of environmental surfaces is an essential way to prevent hospital infection [143]. The potential use of EW in the disinfection of inanimate surfaces have been evaluated experimentally [118,144,145]. Meakin et al. revealed that EW exerts a more effective bacterial kill on door hand, lavatory and seat compared to quaternary ammonium disinfectant [146].

## 8. Future Perspectives

The COVID-19 pandemic has placed an immense burden on healthcare systems and economies around the world. At the time of the study, there was no effective approved vaccine and drug against SARS-CoV-2 available. With increasing hygiene and safety challenges, electrolyzed water holds a potential significance for clinical fields since disinfecting is a critical step during cutting off route transmission [147]. Researchers reported that EW was effective at inactivating SARS-CoV-2, porcine reproductive and respiratory syndrome virus (PRRSV), pseudorabies virus (PRV), foot-and-mouth disease virus (FMDV), Newcastle disease virus [24,148,149,150]. Microorganisms can spread from their source to new hosts through direct or indirect contact, in the air, or through vectors [151].

All the EW exhibits strong antimicrobial efficacy in different fields such as food and hard surface as well as agriculture, medical, and dentistry without irritation [144]. EW has been approved by the Japanese, US, and Chinese regulations as a perfect substitute for harmful chemicals and as a novel sustainable and eco-friendly solution for use in the hospitals and at home. In recent years, a continuous growth trend of commercialization of EW has been observed throughout the world. Given the importance of EW, many companies are scrambling to establish and start producing EW products such as Clortech^®^, Avenova^®^, Ecasol™, MicroSafe^®^ and Microcyn^®^. These companies claim to produce EW-based products that have a remarkable antimicrobial effect, while being safe to use around the nose, mouth, and eyes. However, the limitation of EW is that it has not been widely studied, notably for efficacy against multidrug- and extensively drug-resistant Gram-negative bacteria according with World Health Organization priority pathogens list.

SAEW is the most studied EW and has shown its pH-neutral properties. HOCl was found to be nonirritating and non-sensitizing in various animal safety models. The composition of SAEW solution is relatively simple, and once it becomes exposed to the air, the active ingredients will decompose and its sanitizing efficacy drops [152]. Researchers are constantly exploring the mechanism of the EW antimicrobial effect and developing an advanced and dynamic EW production system that is capable of overcoming all the current limitations. In the near future, this powerful lack of antimicrobial resistance and safety makes SAEW a particularly attractive option for surgical wound site antimicrobial activity, especially in cosmetic, eye care and private women’s care.

## 9. Conclusions

EW is an effective disinfectant, with several advantages such as on-the-spot, cheap, environmentally friendly and safety production. Nowadays, with the development of a novel popular type of SAEW, some limitations have been resolved. It has been reported that SAEW does not irritate the hands, skin, and mucous membranes, and causes no safety issues from Cl_2_ off-gassing. It recently emerged with great potential for clinical applications. However, the antimicrobial effect of EW is influenced by the presence of organic matter, water pollutants, and the hardness of the product. Therefore, a dynamic and advanced EW production system or the hurdle technology of combing with multiple technologies-based EW that are able to overcome currently limitations. These may expand the use of EW in clinical applications.

## Figures and Tables

**Table 1:** Criteria of EW in different countries.

	Japan [101,102,103]	The United States [51]	EU [104]	China [95,96]
Administration	Ministry of Health, Labor, and Welfare	Administration of US Food and Drug	European Commission Directorate-General for Agriculture and Rural Development	Standardization administration
Application	Strong acid electrolyzed water (pH < 2.7): 20–60 ppm: hand washing in operation, cleaning and disinfection of endoscope and food additives.	Poultry Processing Facilities	Buildings and installations Aquaculture (only in the absence of animals)	Indoor air environment General object surface Medical equipment Surface of secondary water supply equipment and facilities
	Slightly acid electrolyzed water (2.7–5.0): 10–60 ppm: food additives and designation of specified pesticides (specific control materials)	Meat Processing	In general agriculture and in organic farming Plant and animal production Food processing	Vegetables and fruits
	Slightly acid electrolyzed water (ph:5.0–6.0): 10–80 ppm: food additives	Fruit and Vegetable Processing Facilities		Fabric
		Fish and Seafood Processing		Utensils
		Processed and Preformed Meat and Poultry		Hands
		Shell Egg Wash Organic Production and Handling		Skin and mucous membrane
ACC concentration	Strong acid electrolyzed water (ph < 2.7): 20–60 ppm Slightly acid electrolyzed water (2.7–5.0): 10–60 ppm Slightly acid electrolyzed water (pH:5.0–6.0): 10–80 ppm	<60 ppm Organic production and Handling(≤4 ppm)	Electrolyzed water usually contains 20–60 ppm (hypochlorite and hypochlorous acid, in a pH-dependent equilibrium).	Requirement of different application of toxicity
Requirement	Electrolyzed water must be decomposed or removed before completion of the final food	The treatment will be followed by either a 10 min drain step or a potable water rinse to remove		Non toxicity

**Table 2:** Characteristics and parameters of various electrolyzed waters.

Type of EW	Diaphragm Electrolyzer	Electrolyte	pH	ORP (mV)	ACC
Acidic electrolyzed water/electrolyzed oxidizing water	Two-cell chambers /anode Three-cell chambers/anode	NaCl water (<0.2%)	2–2.7	>1100	20–60
Weak acid electrolyzed water	Two-cell chambers Three-cell chambers	NaCl water (<0.2%)	2.7–5.0	-	10–60
Slightly acid electrolyzed water	Single-cell chamber (without diaphragm)	HCl water (2–6%)/ The mixture water of NaCl and HCl	5–6.5	850	10–80
Neutralized electrolyzed water	Single-cell unit (without diaphragm)	NaCl or HCl	7–8	750–900	30–200
Alkaline electrolyzed water	Two-cell chambers /cathode	NaCl water	10–13	−800–900	80–100

**Table 3:** Applications of EW against various microorganisms in clinical infections.

Application	Target	EW Type (Product)	Exposure Time	Observations (log CFU)	ACC	pH	ORP (Mv)	Reference
Wound	These comprised three Gram-positive bacteria (*Enterococcus faecium*; *S. epidermidis* and *S. aureus*); three Gram-negative bacteria (*Morganella morganii*; *Enterobacter cloacae* and *P. aeruginosa*) and two yeasts (*Candida albicans* and *Torulopsis glabrata*).	EW Clortech^®^	5	4.57 log CFU/cm^2^	500	-	-	[105]
Eye	*S. epidermidis* colony-forming units	EW Avenova^®^	20	>99.5%	100	4	-	[106]
Wound	*X Pseudomonas* *Staphylococcus aureus*	Slightly acid electrolyzed water (SAEW) Vashe Wound Solution	-	3.78 log/g 4.44 log/g	-	5.5	-	[107]
Atopic dermatitis on skin	*Staphylococcus aureus*	Acidic electrolyzed water (AEW)	3 min after spraying (*P* < 0.05) and after 1 week of skin treatment	3.80 log/cm^2^	-	≤2.7	1000≥	[108]
Wound healing	Hairless mice (wound size)	Slightly acid electrolyzed water (SAEW)	Hairless mice three times a day for seven days	Wound size reduced to 22.4%	25	5.5–6.5	800	[109]
Wound healing	*Pseudomonas aeruginosa*-infected wounds	Weakly acidic hypochlorous acid	Cleansing effects of HOCl and covering with CNFS/Ag NP composites daily for 3 days	Wound size reduced to 23%	200	6.5	-	[110]
Inner layer dentin	The time dependent microhardness values at 25 μm depth	AEW	15 min	75% decrease	49	2.4	-	[111]
Wound biofilms	*S. aureus* biofilms *A. baumannii* biofilms *P. aeruginosa* biofilms	EW	180 120 60	100% 100% 100%	892 524 367	6.0	-	[112]
Wound biofilm	*Staphylococcus aureus* biofilm in vitro *Pseudomonas aeruginosa* biofilm in vitro *Pseudomonas aeruginosa* biofilm in an ex vivo porcine skin explant model	Microcyn^®^	15	4.3 log_10_ CFU/mL reduction 7 log_10_ CFU/mL reduction 0.77 log_10_ CFU /mL reduction	-	-	-	[113]
Atopic dermatitis	NC/Nga mouse model of Atopic dermatitis	EW	Twice a day	less skin lesions prevent scratching bouts nontoxicity	500	6.0	-	[97]
Wound healing	Cytotoxicity in L929 mice fibroblast cells Wound healing activity	Strong acid electrolyzed water (StAEW)	Scratch assay	88.84% wound healing ratio No mutagenic activity	32.87	2.4	1140.67	[114]
Oral Pathologic Bacteria Species	*A. actinomycetemcomitans* *S. salivarius* *L. casei* *S. aureus*	AEW	0.5	100% 99.92% 99.99% 98.04%	-	3	-	[115]
Dental plaque (biofilm)	*Streptococcus mutans* biofilm	SIEW		3 log reduction CFU/cm^2^	5	11.4–11.7	−868	[116]
Ascetic fluid	Surgical site infection including *Escherichia coli*, *Bacteroides fragilis*, γ-*hemolytic Streptococcus*)	StAEW	-	No one infection in 24 patients	40	2.5–2.7	1000–11000	[117]
Titanium alloy surfaces	*E. coli* *P. gingivalis* *E. faecalis* *S. sanguinis*	EW	1.5	100% 100% 100% 100%	180	5.5	-	[118]
Toothbrushes	*A.actinomycetemcomitans* *F. nucleatum* *P. intermedia* *P. gingivalis*	EW	0.5	11.0–12.4%	30	8.4	-	[119]
Oral comprehensive treatment table	*Pseudomonas aeruginosa* and *Legionella pneumophila*	SAEW	Flush the oral comprehensive treatment table	4.30 log/mL	10	5.5–6.5	982	[120]
Floor, table, mattress, sheet, blanket, curtain	*Escherichia coli* *Staphylococcus aureus* *Enterococcus faecalis* *Pseudomonas aeruginosa* *Aspergillus fumigatus* *Acinetobacter baumannii* *Clostridium difficile*	Ecasol™	1.5 h	≥7 log/cm^2^	1000	Ph neutral	-	[121]
Oral bacteria strains	*Porphyromonas gingivalis* *Prevotella intermedia* *Prevotella nigrescens* *Fusobacterium nucleatum* *Streptococcus mutans* *Streptococcus sobrinus* *Streptococcus gordonii* *Streptococcus oralis* *Streptococcus salivarius*	SAEW	1	≥99.999% ≥99.999% ≥99.9999% ≥99.9999% ≥99.9999% ≥99.999% ≥99.99% ≥99.99999% ≥99.9999%	3–5	5–7	-	[122]
Porous	Noroviruses	EW	10	3 log/cm^2^	200	5.5–6.2	-	[123]
